# Mesothelin is a target of chimeric antigen receptor T cells for treating gastric cancer

**DOI:** 10.1186/s13045-019-0704-y

**Published:** 2019-02-18

**Authors:** Jiang Lv, Ruocong Zhao, Di Wu, Diwei Zheng, Zhiping Wu, Jingxuan Shi, Xinru Wei, Qiting Wu, Youguo Long, Simiao Lin, Suna Wang, Zhi Wang, Yang Li, Yantao Chen, Qing He, Suimin Chen, Huihui Yao, Zixia Liu, Zhaoyang Tang, Yao Yao, Duanqing Pei, Pentao Liu, Xuchao Zhang, Zhenfeng Zhang, Shuzhong Cui, Ren Chen, Peng Li

**Affiliations:** 1 Key Laboratory of Regenerative Biology, South China Institute for Stem Cell Biology and Regenerative Medicine, Guangzhou Institutes of Biomedicine and Health, Chinese Academy of Sciences, Guangzhou, China; 2 Guangdong Provincial Key Laboratory of Stem Cell and Regenerative Medicine, South China Institute for Stem Cell Biology and Regenerative Medicine, Guangzhou Institutes of Biomedicine and Health, Chinese Academy of Sciences, Guangzhou, China; 3 University of Chinese Academy of Sciences, Shijingshan District, Beijing, China; 4 School of Life Sciences, University of Science and Technology of China, Hefei, China; 5 The Center of Research Animal, Guangzhou Institutes of Biomedicine and Health, Chinese Academy of Sciences, Guangzhou, 510530 China; 6 Department of Pediatric Hematology/Oncology, Sun Yat-Sen Memorial Hospital, Sun Yat-Sen University, Guangzhou, China; 7 Orthopaedics Department, Sun Yat-Sen Memorial Hospital, Sun Yat-Sen University, Guangzhou, 510120 China; 8 SICU Department, Sun Yat-Sen Memorial Hospital, Sun Yat-Sen University, Guangzhou, 510120 China; 9 Huangpu Hospital of Guangdong Second Traditional Chinese Medicine Hospital, Guangzhou, 510120 China; 10 Department of Outpatient, The 91th Military Hospital, Jiaozuo, China; 11 Division of Reproductive Endocrinology, The 91th Military Hospital, Jiaozuo, China; 12 Guangdong Zhaotai InVivo Biomedicine Co. Ltd., Guangzhou, China; 13 School of Biomedical Sciences, Li Ka Shing Faculty of Medicine, Stem Cell and Regenerative Medicine Centre, University of Hong Kong, Hong Kong, China; 14 Guangdong Lung Cancer Institute, Medical Research Center, Guangdong General Hospital, Guangdong Academy of Medical Sciences, Guangzhou, China; 15 Department of Radiology, The Second Affiliated Hospital of Guangzhou Medical University, Guangzhou, China; 16 Affiliated Cancer Hospital & Institute of Guangzhou Medical University, Guangzhou, China; 17 Department of Infectious Disease, Guangdong General Hospital, Guangdong Academy of Medical Sciences, Guangzhou, China; 18 Hefei Institute of Stem Cell and Regenerative Medicine, Guangzhou Institutes of Biomedicine and Health, Chinese Academy of Sciences, Guangzhou, 510530 China

**Keywords:** Gastric cancer, Mesothelin, Chimeric antigen receptor T cells, Immunotherapy, Immunodeficient mice

## Abstract

**Background:**

Gastric cancer (GC) is a common cancer in Asia and currently lacks a targeted therapy approach. Mesothelin (MSLN) has been reported to be expressed in GC tissue and could be targeted by chimeric antigen receptor (CAR) T cells. Mesothelin targeting CAR-T has been reported in mesothelioma, lung cancer, breast cancer, and pancreas cancer. However, the feasibility of using anti-MSLN CAR T cells to treat GC remains to be studied.

**Methods:**

We verified MSLN expression in primary human GC tissues and GC cell lines and then redirected T cells with a CAR containing the MSLN scFv (single-chain variable fragment), CD3ζ, CD28, and DAP10 intracellular signaling domain (M28z10) to target MSLN. We evaluated the function of these CAR T cells in vitro in terms of cytotoxicity, cytokine secretion, and surface phenotype changes when they encountered MSLN+ GC cells. We also established four different xenograft GC mouse models to assess in vivo antitumor activity.

**Results:**

M28z10 T cells exhibited strong cytotoxicity and cytokine-secreting ability against GC cells in vitro. In addition, cell surface phenotyping suggested significant activation of M28z10 T cells upon target cell stimulation. M28z10 T cells induced GC regression in different xenograft mouse models and prolonged the survival of these mice compared with GFP-transduced T cells in the intraperitoneal and pulmonary metastatic GC models. Importantly, peritumoral delivery strategy can lead to improved CAR-T cells infiltration into tumor tissue and significantly suppress the growth of GC in a subcutaneous GC model.

**Conclusion:**

These results demonstrate that M28z10 T cells possess strong antitumor activity and represent a promising therapeutic approach to GC.

## Background

Gastric cancer (GC) is one of the most common cancer types worldwide and is the second leading cause of cancer-related death in Asia [1]. Currently, the 5-year survival rate of GC patients is approximately 20–30%. Most patients are diagnosed with advanced stage or metastatic disease and therefore are not candidates for surgery [2–4]. Although overall survival and quality of life are improved by chemo- and radiotherapeutic approaches, the median overall survival remains less than 12 months for patients diagnosed with advanced stage GC [3, 5]. Recently, targeted strategies, including HER-2 and VEGFR-2 monoclonal antibodies, have achieved substantial responses in GC patients [5–10]. Meanwhile, immune checkpoint inhibitors, such as antibodies against CTLA-4, PD-1, or PD-L1, are being investigated in different phase clinical trials [11–14]. Nonetheless, the response rate and progression-free survival remain poor for patients with advanced GC.

Chimeric antigen receptor (CAR) T cell therapy has been developed for the treatment of different types of cancer, including acute lymphoblastic leukemia and various solid cancers [15–20]. CARs are composed of an extracellular single-chain variable fragment (scFv) that recognizes diverse tumor-associated antigens (TAAs), a transmembrane fragment, a CD3ζ T cell activating domain, and a costimulatory domain, such as the intracellular domains of CD28 and 4-1BB [21–24]. Upon specific binding of CAR to a TAA on target tumor cells, the CD3ζ and costimulatory domains are activated, and the phosphorylation cascade is triggered in T cells, leading to the release of cytotoxic granules, the transcription of genes encoding cytokines, and cell proliferation [25].

Mesothelin (MSLN) is a 40-kDa membrane protein that has been reported to be expressed on normal mesothelial tissue and highly expressed in mesothelioma and lung, pancreas, breast, ovarian, and gastric cancer [26–28]. To date, MSLN has been utilized as a target of CAR T cells against solid cancers, including mesothelioma, lung cancer, breast cancer, and pancreas cancer [29–33]. However, the feasibility of using anti-MSLN CAR T cells to treat GC remains to be explored. In the previous studies, we constructed third-generation CAR M28z10 and demonstrated the improved anti-tumor activities compared with the second-generation CAR M28z against lung cancer [34]. In the present study, we firstly identified MSLN as an available target on GC tissue and cells and then characterized the efficacy of anti-MSLN CAR (M28z10) T cells against GC in multiple in vitro functional assays and in vivo xenograft mouse models, and we proved the improved CAR-T cell infiltration and efficacy by regional peritumoral delivery compared with systemic intravenous delivery approach. Our findings demonstrate that M28z10 T cells possess strong antitumor activity against GC and pave the way for the future clinical application of this treatment for GC patients.

## Materials and methods

### Chimeric antigen receptor vector design

The third-generation anti-MSLN CARs containing both CD28 and DAP10 cytoplasmic sequences were previously reported [34, 35]. Briefly, human DAP10 cytoplasmic domain sequence was obtained from the UniProt database (ID: Q9UBK5). The third-generation anti-MSLN containing the DAP10 cytoplasmic sequence were synthesized by Genscript (Nanjing) Co., Ltd. (Nanjing, China), and cloned into the second-generation lentiviral vector pWPXLd-2A-eGFP through Pme1 and Spe1 cloning sites.

### Lentivirus production

Lentivirus particles were produced in HEK-293 T cells via polyethyleneimine (Sigma-Aldrich, St Louis, MO, USA) transfection. The pWPXLd-based lentiviral plasmid and two packaging plasmids, psPAX2 and pMD.2G, were cotransduced into HEK-293 T cells in 10 cm dish at a ratio of 3:1:4, with a total amount of 24 μg. Lentivirus-containing supernatants were harvested at 24, 48, and 72 h posttransfection and filtered through a 0.45-μm filter.

### Isolation, transduction, and expansion of primary human T lymphocytes

Peripheral blood mononuclear cells (PBMCs) were isolated from the buffy coats of healthy donors using Lymphoprep (Fresenius Kabi Norge, AS, Berg i Østfold, Norway). T cells were negatively selected from PBMCs using a MACS Pan T Cell Isolation Kit (Miltenyi Biotec, Bergish Gladbach, Germany) and activated using microbeads coated with anti-human CD3, anti-human CD2, and anti-human CD28 antibodies (Miltenyi Biotec) at a 1:1 bead:cell ratio for 24 h in RPMI-1640 supplemented with 10% heat-inactivated fetal bovine serum (FBS), 40 IU/ml interleukin-2 (IL-2), 10 mM HEPES, 2 mM glutamine, and 1% penicillin/streptomycin. Every 1*10^6^ T cells were transduced with 5–10 ml CAR lentiviral supernatants in the presence of 8 μg/ml polybrene (Sigma) for 5 h with 1 ml 10% FBS containing RPMI1640, and a continuous two rounds of transduction were conducted. After transduction, T cells were cultured in fresh media containing IL-2 (300 IU/ml). Subsequently, fresh media were added every 2–3 days to maintain cell density within the range of 0.5–1 × 10^6^/ml. Healthy PBMC donors who provided primary specimens gave informed consent for the use of their samples for research purposes, and all procedures were approved by the Research Ethics Board of Guangzhou Institutes of Biomedicine and Health (GIBH).

### Cells and culture conditions

HEK-293 T cells were maintained in Dulbecco’s modified Eagle medium (Gibco, Grand Island, NY, USA). K562 (human myelogenous leukemia cell line), AGS (human gastric adenocarcinoma), BGC-823 (human gastric adenocarcinoma), KATO III (human gastric carcinoma), and MKN-28 (human gastric carcinoma) cell lines were obtained from ATCC (Manassas, VA, USA) and maintained in RPMI-1640. Luciferase/GFP-expressing cell lines (K562-GL, AGS-GL, BGC-823-GL, KATO III-GL, MKN-28-GL) were generated by transfection of the parental cell line with lentiviral supernatant containing luciferase-2A-GFP and were sorted for GFP expression on a FACS AriaTM cell sorter (BD Biosciences, San Jose, CA, USA). DMEM and RPMI-1640 media were supplemented with 10% heat-inactivated FBS (Gibco/Life Technologies), 10 mM HEPES, 2 mM glutamine (Gibco/Life Technologies), and 1% penicillin/streptomycin. All cells were cultured at 37 °C in an atmosphere of 5% carbon dioxide.

### Flow cytometry

All samples were analyzed using a NovoCyteTM (ACEA Biosciences), LSR Fortessa, or C6 flow cytometer (BD Biosciences), and data were analyzed using FlowJo software (FlowJo, LLC, Ashland, OR, USA). The antibodies used included anti-MSLN-biotin (clone MB), Streptavidin-APC, anti-human CCR7-APC (clone 3D12), anti-human CD62L-PE (clone DREG-56), anti-human CD45RA-APC (clone HI100), anti-human CD45RO-PE (clone UCHL1), anti-human TIM3-PE (clone F38-2E2), anti-human LAG3-PerCP/Cy5.5 (clone 11C3C65), anti-human PD-1-APC (clone NAT105), anti-human CD27-PE (clone M-T271), anti-human CD28-APC (clone CD28.2), anti-human CD25-PE (clone BC96), anti-human CD69-APC (clone FN50), anti-human CD107a-APC (clone H4A3), anti-human CD3-APC (clone UCHT1), anti-human CD4-PerCP/Cy5.5 (clone OKT4), anti-human CD8-PE (clone OKT8), mouse IgG2a isotype control-APC (clone RMG2a-62), mouse IgG1kappa isotype control-PE, mouse IgG1kappa isotype control-PerCP/Cy5.5, and mouse IgG1kappa isotype control-APC (clone MOPC-21) (Biolegend, San Diego, CA, USA). All FACS-related staining procedures were performed on ice for 30 min, and cells were then washed with PBS containing 1% FBS before cytometry analysis. PB, spleen (SP), and tumor samples from mouse xenografts were treated with red blood cell lysis buffer (Biolegend), and the cells were stained with the corresponding antibodies.

### In vitro tumor killing assays

AGS-GL, BGC-823-GL, KATO III-GL, and MKN-28-GL target cells were incubated with GFP or CAR-MSLN T cells at the indicated ratio in triplicate wells of white 96-well plates. Target cell viability was monitored 18 h later by adding 100 μl/well d-luciferin (potassium salt) (Cayman Chemical, USA) at 150 μg/ml. Background luminescence was negligible (< 1% of the signal from wells containing only target cells). The percent viability (%) was calculated as experimental signal/maximal signal × 100, and the percent lysis was equal to 100% viability.

### Cytokine release assays

Enzyme-linked immune absorbance assay (ELISA) kits for IL-2, interferon-γ (IFN-γ), granzyme B, and granulocyte-macrophage colony-stimulating factor (GM-CSF) were purchased from eBioscience (San Diego, CA, USA), and all ELISAs were performed according to the manufacturer’s protocols. T cells were cocultured with target cells at an E:T ratio of 1:2 for 18 h. The culture supernatants were then collected and analyzed for the secretion of IL-2, IFN-γ, GM-CSF, and granzyme B using ELISA kits.

### CDX models for CAR T cell treatment

Animal experiments were performed in the Laboratory Animal Center of GIBH, and all animal procedures were approved by the Animal Welfare Committee of GIBH. All protocols were approved by the relevant Institutional Animal Care and Use Committee (IACUC). All mice were maintained in specific pathogen-free (SPF)-grade cages and were provided autoclaved food and water.

For the intraperitoneal GC models, 1 × 10^6^ BGC-823-GL cells in 100 μl PBS were injected into the peritoneal cavity of NSI mice aged 6–8 weeks. Two weeks after tumor cell injection, the mice were subjected to bioluminescence imaging (BLI) and randomly divided into three groups: NC, GFP-T, and M28z10-T. Mice received 5 × 10^6^ M28z10-T cells or equivalent number of GFP-T cells suspended in 100 μl PBS by tail vein injection. On day 21 and 33, mice were subjected to the BLI analysis again.

For the pulmonary metastatic GC models, 1 × 10^6^ BGC-823-GL cells in 100 μl PBS were injected into NSI mice aged 6–8 weeks through tail vein. Two weeks after tumor cells injection, the mice were subjected to BLI and randomly divided into two groups: GFP-T and M28z10-T. Mice received 5 × 10^6^ M28z10-T cells and equivalent number of GFP-T cells suspended in 100 μl PBS and injected into the mice through tail vein. On day 23 and 35, mice were subjected to BLI analysis again.

For the cell line-based GC subcutaneous (s.c.) xenograft models, 1 × 10^6^ BGC-823 or MKN-28 cells in 100 μl PBS were injected subcutaneously into the right flanks of NSI mice aged 6–8 weeks. When tumor nodes were palpable, the mice were divided into five groups (NC, GFP-T i.v., GFP-T p.t., M28z10-T i.v., and M28z10-T p.t.) and received 5 × 10^6^ M28z10-T cells or the equivalent number of GFP-T cells in 100 μl PBS intravenously or peritumorally. Tumor volume were measured twice a week with a caliper and calculated by the following equation: tumor volume = (length × width^2^)/2. For the CAR-T cell infiltration experiment, 1 × 10^6^ BGC-823 cells in 100 μl PBS were injected s.c. into the right flanks of NSI mice aged 6–8 weeks. When tumor nodes were palpable, mice were randomly divided into two groups (i.v. and p.t., *n* = 18) and received 5 × 10^6^ M28z10-T cells in 100 μl PBS intravenously or peritumorally. On day 3, 9, and 15 after M28z10-T cells injection, six mice were sacrificed and tumor tissues was dissected to examine the status of CAR-T cells infiltration by FACS and immunofluorescence imaging respectively.

### Bioluminescence imaging

In vivo whole-body imaging of luciferase-labeled cells was performed using a cooled CCD camera system (IVIS 100 Series Imaging System, Xenogen, Alameda, CA, USA). d-Luciferin firefly potassium salt was injected at 75 mg/kg. Mice were anesthetized by isoflurane and subject to imaging 5 min after the injection of substrate. Quantification of total and average emissions was performed using Living Image software (Xenogen).

### Immunohistochemistry and immunofluorescence imaging

Tumor tissue sections were fixed with 10% paraformaldehyde, embedded in paraffin, sectioned at a thickness of 4 μm, and stained using a standard hematoxylin and eosin technique. Paraffin sections were also immunostained with antibodies specific for MSLN (ZSGB-BIO, Beijing, China) overnight at 4 °C, followed by secondary staining with goat anti-rabbit Ig (PV-9000) (ZSGB-BIO, Beijing, China). Images of all sections were obtained with a microscope (DMI6000B; Leica Microsystems, Wetzlar, Germany).

For the immunofluorescence imaging, paraffin sections were incubated with antibodies specific for CD3 (ZSGB-BIO, Beijing, China) overnight at 4 °C, followed by secondary staining with goat anti-rabbit IgG (H + L) (Beyotime, Shanghai, China). Images of sections were obtained with a laser scanning confocal microscopy (LSM 800, Carl Zeiss AG, Oberkochen, Germany).

### Statistics

Data are presented as the means ± standard errors of the means. Student’s *t* test was used to determine the statistical significance of differences between samples, and a *P* value < 0.05 indicated a significant difference. All statistical analyses were performed using Prism software, version 7.0 (GraphPad, Inc., San Diego, CA, USA).

## Results

1. MSLN expression in primary GC tissue and cell lines

**Fig. 1:**
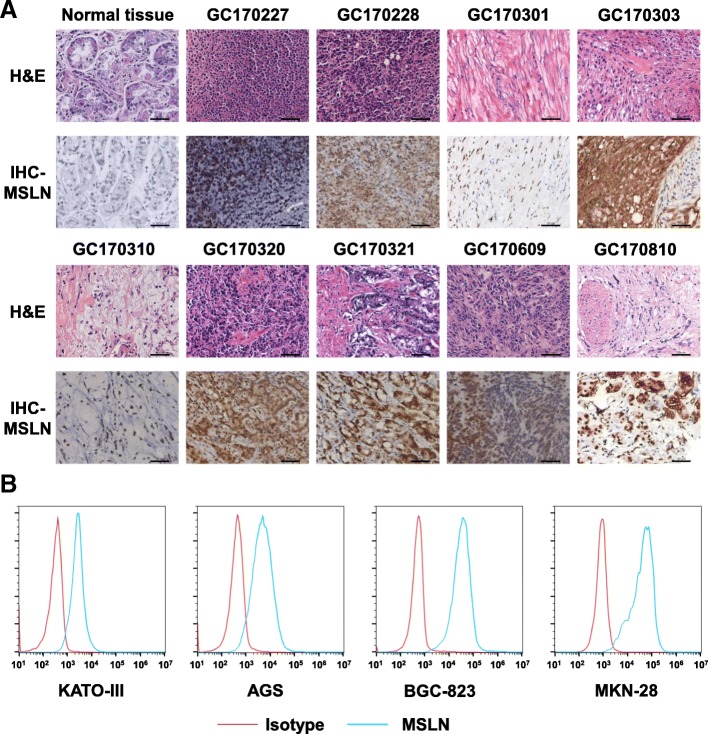
MSLN expression in primary GC tissues and cell lines. **a** Immunohistochemical staining for MSLN in normal gastric tissue and nine primary GC samples, scale bar = 100 μm. **b** Detection of MSLN expression in four human GC cell lines, including KATO III, AGS, BGC-823, and MKN-28 cells, by flow cytometry

Tumor targeting by CAR T cells requires the expression of certain TAAs on the surface of tumor cells. To evaluate MSLN expression in primary GC tissue, we performed immunohistochemical staining for MSLN in nine primary GC samples and found robust expression in most of these samples compared with normal gastric tissue (Fig. 1a). We examined MSLN expression in four human GC cell lines, including BGC-823, AGS, KATO III, and MKN-28 cells, by flow cytometry. All four cell lines expressed MSLN, but BGC-823 and MKN-28 cells expressed higher levels than did AGS and KATO III cells (Fig. 1b). Collectively, these results indicate that MSLN expression is upregulated in both GC primary cells and cell lines.

2. Generation of third-generation CAR T cells targeting MSLN

**Fig. 2:**
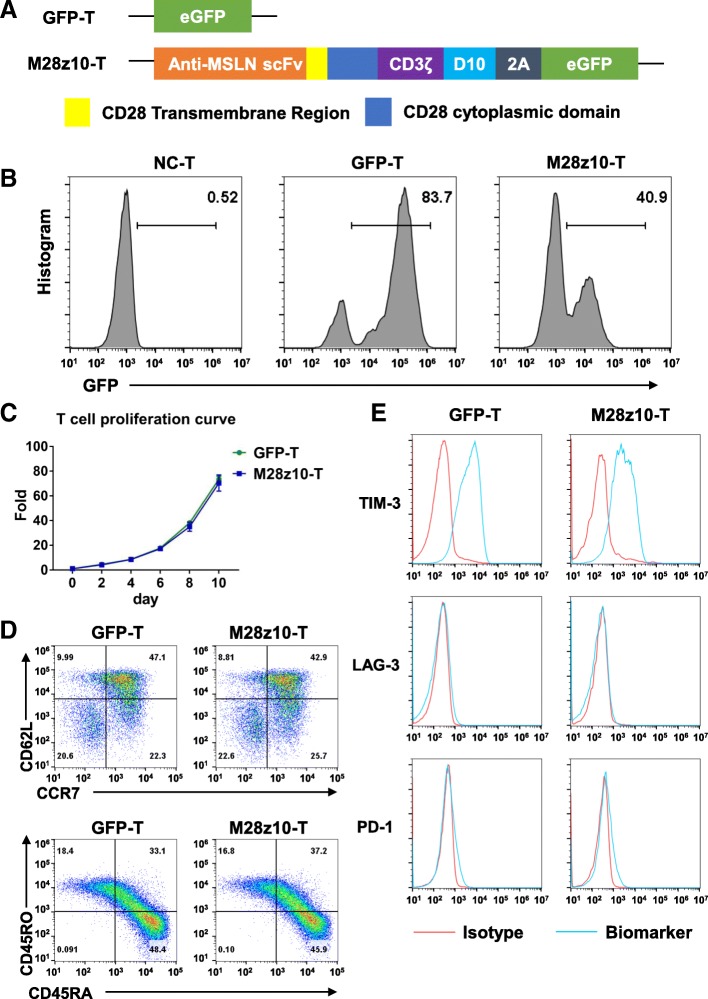
Generation of third-generation CAR T cells targeting MSLN. **a** Schematic diagram of the M28z10 transgene. **b** Percentage of GFP and M28z10 transduced primary human T cells detected by flow cytometry. **c** Representative graph of the expansion rate of M28z10 CAR T cells in 10 days. **d** Detection of CCR7, CD62L, CD45RA, and CD45RO on the manufactured T cells. **e** Detection of exhaustion markers, including TIM-3, LAG-3, and PD-1 on the manufactured T cells

To redirect human T cells to the MSLN antigen expressed by GC tumor cells, we constructed the third-generation M28z10 vector containing the scFv that recognizes MSLN, CD28 transmembrane domain, CD3ζ T cell activating domain, and the costimulatory domains from both CD28 and DAP10 as previously described [23, 36]. CAR was coexpressed with eGFP separated by a 2A sequence (Fig. 2a). Primary human T lymphocytes isolated from peripheral blood mononuclear cells (PBMCs) by magnetic selection were activated with anti-CD3/CD28/CD2-coated beads for 24 h before transduction with the M28z10 transgene. Transduction efficiency was determined after 72 h by the percentage of GFP+ cells detected by flow cytometry (Fig. 2b). The transduced T cells were cultured for 10 days, achieving a greater than 60-fold expansion with the addition of 300 IU of exogenous interleukin-2 (IL-2) (Fig. 2c). GFP-transduced T cells were used as a control group. A substantial fraction of manufactured CAR T cells showed a CD45RA^+^CCR7^+^CD62L^high^ phenotype. Most of the cells express TIM-3, but expression levels of PD-1 and LAG-3 are pretty low as detected by FACS (Fig. 2d, e).

3. M28z10 T cells showed strong antitumor activity against GC cell lines in vitro

**Fig. 3:**
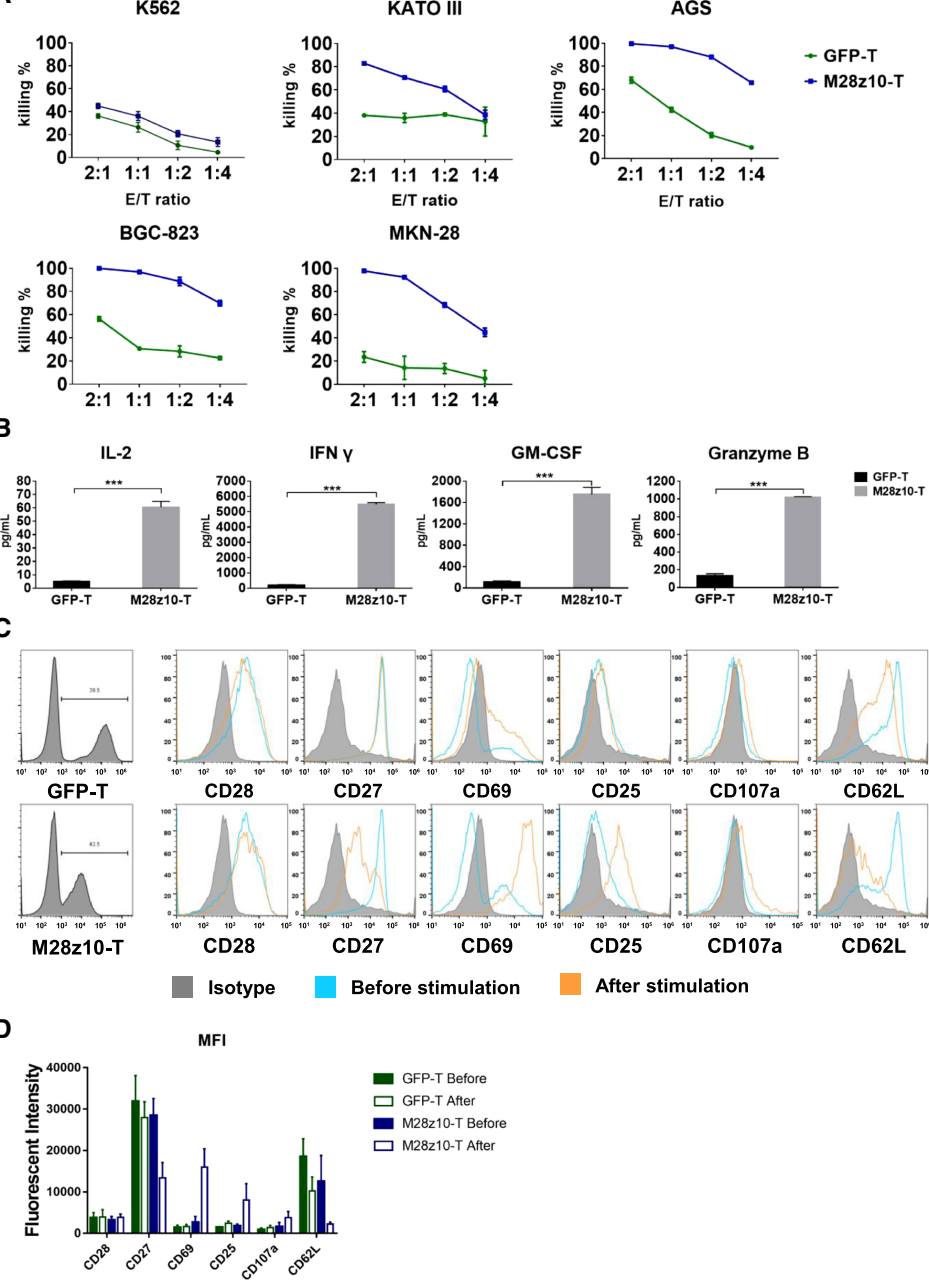
M28z10 T cells showed strong antitumor activity against GC cell lines in vitro. **a** Eighteen-hour in vitro killing assays of M28z10 T cells and GFP T cells in multiple GC cell lines, including K562-GL, KATO III-GL, AGS-GL, BGC-823-GL, and MKN-28-GL cells, at each E:T ratio. **b** Detection of IL-2, IFN-γ, GM-CSF, and granzyme B secretion by M28z10 and GFP T cells after coculture with BGC-823-GL cells for 18 h at an E:T ratio of 1:2. Error bars denote the s.e.m., and the results were compared with an unpaired *t* test. *P < 0.05, **P < 0.01, and ***P < 0.001. **c** Detection of multiple cell surface markers on GFP and M28z10 T cells after stimulation with BGC-823 cells at an E:T ratio of 1:2 or no stimulation (gated on CD3 and GFP double positive cells). **d** Statistical analysis of three independent FACS results. Error bars denote the s.e.m.

To determine the cytotoxicity of M28z10 T cells against MSLN+ GC cell lines in vitro, we lentivirally transduced 5 GC cell lines with a GFP-2A-luciferase transgene and thereby constructed the KATO III-GL, AGS-GL, BGC-823-GL, and MKN-28-GL cell lines. Cell viability was determined by a luciferase reporter system and an illuminator [37]. We performed an 18-h killing assay of M28z10 T and GFP T cells on the four GC cell lines along with K562-GL cells which were used as a mesothelin negative control. The results showed that M28z10 T cells exerted stronger cytotoxicity than GFP T cells after coculture with all four GC cell lines at the indicated effector to target (E:T) ratio in vitro, while for mesothelin negative K562-GL cells, cytotoxicity remain almost same between M28z10 and GFP T cells (Fig. 3a). To analyze cytokine secretion by M28z10 T cells after target cell stimulation, we collected the culture supernatant of BGC-823-GL tumor cells in a killing assay and then detected cytokines and effector molecules that are generally secreted by activated T cells, including IL-2, interferon-γ (IFN-γ), granulocyte-macrophage colony-stimulating factor (GM-CSF), and granzyme B, by enzyme-linked immune absorbance assay (ELISA). There was a significant increase in the secretion of these 4 proteins by M28z10 T cells compared with GFP T cells (Fig. 3b), indicating the strong cytokine-secreting capabilities of M28z10 T cells upon encountering MSLN+ GC cells.

T cell activation and differentiation can be monitored based on changes in the expression of a series of surface biomarkers, such as CD25, CD28 and CD69 (generally accepted biomarkers of T cell activation), and CD27 and CD62L (biomarkers positively related to the memory phenotype). In addition, surface CD107a expression positively correlates with T cell degranulation, a key process responsible for cytotoxicity against target cells. We then performed flow cytometry to determine the levels of these biomarkers on M28z10 and GFP T cells after stimulation with BGC-823 target tumor cells. The results suggested the upregulation of CD25, CD69, and CD107a and the downregulation of CD27 and CD62L on M28z10 T cells after stimulation with BGC-823 cells compared with unstimulated cells and GFP T cells. Additionally, CD28 levels remained almost unchanged in these groups of cells (Fig. 3c, d). Collectively, our findings demonstrated that in contrast to GFP T cells, M28z10 T cells exhibited robust cytotoxicity and cytokine production as well as marked changes in the surface phenotype after they encountered target MSLN+ tumor cells in vitro, which suggests that M28z10 T cells can elicit strong immune responses and antitumor activity against GC tumor cells.

4. M28z10 T cells showed strong antitumor activity against GC in vivo in both intraperitoneal and pulmonary metastatic GC models

**Fig. 4:**
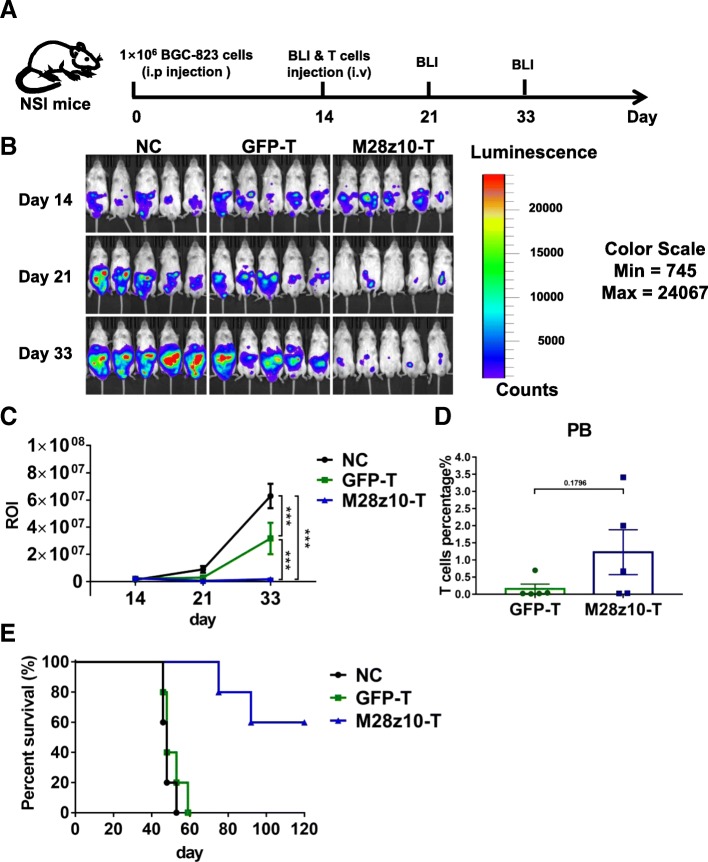
M28z10 T cells showed strong antitumor activity in vivo in an i.p. GC model. **a** Schematic representation of the experiments. **b** BLI of BGC-823-GL intraperitoneally injected mice treated with GFP T or M28z10 T cells. NSI mice received an i.p. injection of 1 × 10^6^ BGC-823-GL cells. After 14 days, 5 × 10^6^ M28z10 T cells or the equivalent number of GFP T cells were injected through the tail vein. On days 14, 21, and 33, BLI was conducted. **c** Statistical analysis of the ROI of each BLI at each time point. Error bars denote the s.e.m., and the results were compared with two-way ANOVA test. *P < 0.05, **P < 0.01, ***P < 0.001. **d** Percentage of T cells in the PB of BGC-823-GL intraperitoneally injected mice. Error bars denote the s.e.m., and the results were compared with an unpaired *t* test. *P < 0.05; **P < 0.01; ***P < 0.001. **e** Survival curve of BGC-823-GL intraperitoneally injected mice. The results were performed with Log-rank (Mantel-Cox) test. *P* value = 0.0014

Our in vitro results suggest that M28z10 T cells can be activated by MSLN+ GC cells and that they possess strong antitumor activity against multiple GC cell lines. To determine the in vivo efficacy of M28z10 T cells against GC, we utilized NOD-SCID IL2Rγ^−/−^ (NSI) immunodeficient mice [36, 38] to establish several different human GC xenograft mouse models. We firstly constructed a tumor model in which 1 × 10^6^ BGC-823-GL GC cells were intraperitoneally (i.p.) injected into the NSI mice. After 14 days, the mice were subjected to bioluminescence imaging (BLI), and robust intraperitoneal expansion of tumor cells was observed (Fig. 4a, b). These mice were then divided into three experimental groups: one, no T cell treatment (blank group); two, GFP-transduced T cell infusion via tail vein injection; and three, injection of 5 × 10^6^ M28z10 T cells. The dosage of GFP T cells in group 2 was the same as the total T cell number in group 3. M28z10 T cells induced significant regression or even elimination of BGC-823-GL GC cells, while tumors in the blank and GFP T groups continued to progress, as detected by BLI (Fig. 4b, c). The persistence of M28z10 T cells was detected in the peripheral blood (PB) at the second week after T cell infusion (Fig. 4d). Intriguingly, long-term survival was observed in most mice in M28z10 T cell-treated group; however, a majority of the mice in the blank and GFP T-treated groups died between 45 and 60 days after the start of the experiment, suggesting that treatment with M28z10 T cells can significantly prolong the survival of tumor-bearing mice (Fig. 4e). Xenogenic graft versus host disease (GVHD) was observed in some of the mice in M28z10 T cell group as they showed significant hair loss, which should be the reason for the decrease of their survival.

**Fig. 5:**
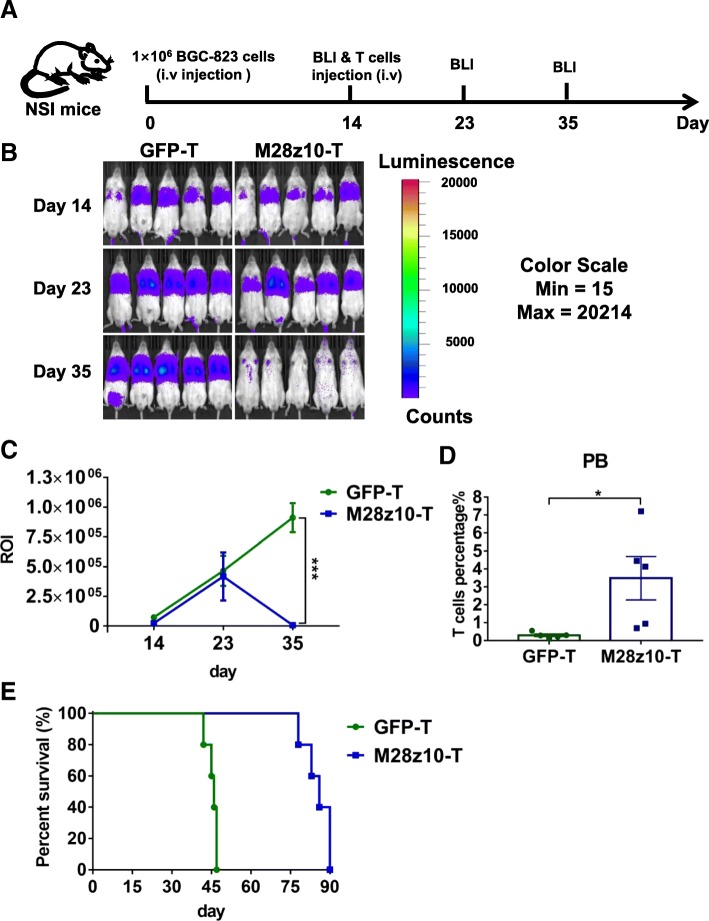
M28z10 T cells showed strong antitumor activity in vivo in a pulmonary metastatic GC model. **a** Schematic representation of the experiments. **b** BLI of BGC-823-GL intravenously injected mice treated with GFP T or M28z10 T cells. Briefly, NSI mice received an i.v. injection of 1 × 10^6^ BGC-823-GL cells. After 14 days, 5 × 10^6^ M28z10 T cells or the equivalent number of GFP T cells were injected through the tail vein, and BLI was conducted on days 14, 23, and 35. **c** Statistical analysis of the ROI of BLI at each time point. Error bars denote the s.e.m., and the results were compared with two-way ANOVA test. *P < 0.05; **P < 0.01; ***P < 0.001. **d** Percentage of T cells in the PB of BGC-823-GL intravenously injected mice. Error bars denote the s.e.m., and the results were compared with an unpaired t-test. *P < 0.05; **P < 0.01; ***P < 0.001. **e** Survival curve of BGC-823-GL intravenously injected mice. The results were performed with Log-rank (Mantel-Cox) test. *P* value = 0.0027

To determine if M28z10 T cells can suppress the distant metastasis of GC, we constructed a mouse model in which BGC-823-GL cells were intravenously injected into NSI mice (Fig. 5a). In these models, tumor cells were detected in the lung (Fig. 5b), thus mimicking the pulmonary metastasis of GC. Fourteen days after tumor cell injection, we infused 5 × 10^6^ M28z10 T cells and the same amount of total GFP-transduced T cells into these pulmonary tumor-bearing mice. BLI results demonstrated that M28z10 T cells almost eliminated pulmonary tumor cells in most of the mice in day 35, while GFP-transduced T cells could not control tumor cell progression (Fig. 5b, c). Significantly, higher percentage of T cells was detected in the M28z10 T group at the second week after T cell infusion, indicating the expansion of M28z10 T cells in vivo (Fig. 4d). Finally, pulmonary-colonized tumor cells caused mouse death within 60 days in the GFP T group. In contrast, a majority of the mice in the M28z10 group continued to survive until 80 days (Fig. 5e) and finally died of xenogenic GVHD. Collectively, our data demonstrated that M28z10 T cells exert strong antitumor activity against MSLN^+^ GC cells in vivo.

5. Peritumoral delivery of M28z10 T cells showed significantly improved tumor infiltration and efficacy compared with intravenous delivery approach in subcutaneous GC mouse models

**Fig. 6:**
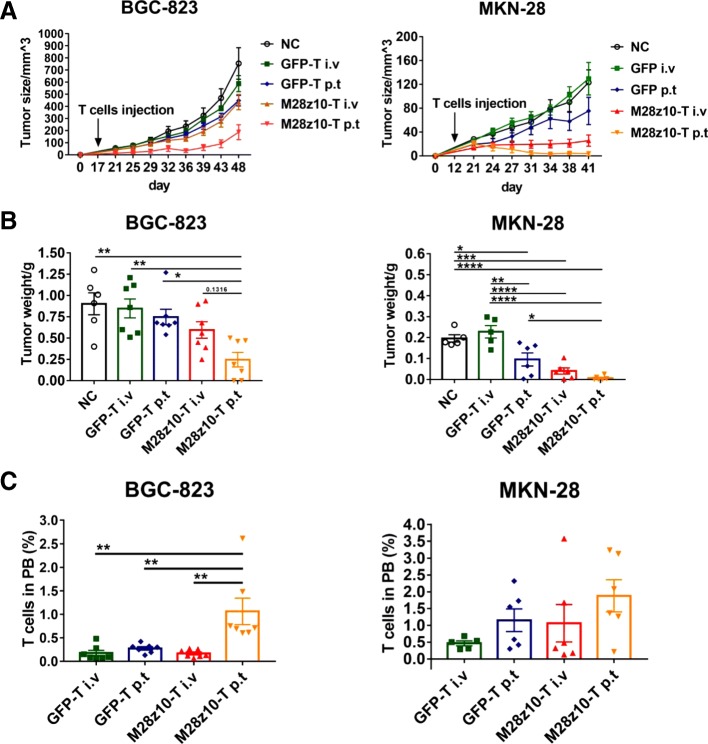
M28z10 T cells showed antitumor activity in vivo in s.c. GC mouse models. **a** Tumor volume of BGC-823 and MKN-28 subcutaneously injected mice. Tumor volume = (length × width^2^)/2. **b** Tumor weight of BGC-823 and MKN-28 subcutaneously injected mice. **c** Percentage of T cells in PB of BGC-823 and MKN-28 subcutaneously injected mice. Error bars denote the s.e.m., and the results were compared with ordinary one-way ANOVA test. *P < 0.05; **P < 0.01; ***P < 0.001

To further verify the efficacy of M28z10 T cells on MSLN+ GC cells in vivo, we established mouse models in which BGC-823 cells or MKN-28 cells were subcutaneously transplanted into NSI mice. When tumor nodules were palpable, mice were treated with GFP-transduced T cells or M28z10 T cells via different delivery methods, including intravenous (i.v.) or peritumoral (p.t.) injection. Thus, the mice were divided into five groups: blank, GFP i.v., GFP p.t., M28z10 i.v., and M28z10 p.t. In total, 5 × 10^6^ M28z10 T cells were injected into the mice in each of the CAR-T groups, and the same number of GFP T cells were injected into the mice of the GFP T cell group (Fig. 6a). The results show that p.t. delivery of M28z10 T cells significantly inhibited the growth of BGC-823 subcutaneous (s.c.) tumors and MKN-28 subcutaneous tumors, while i.v. delivery of M28z10 T cells showed modest efficacy for BGC-823 tumors, but significantly suppressed growth of MKN-28 tumors (Fig. 6a, b). Persistence of M28z10 T cells was detected in PB (Fig. 6c).

**Fig. 7:**
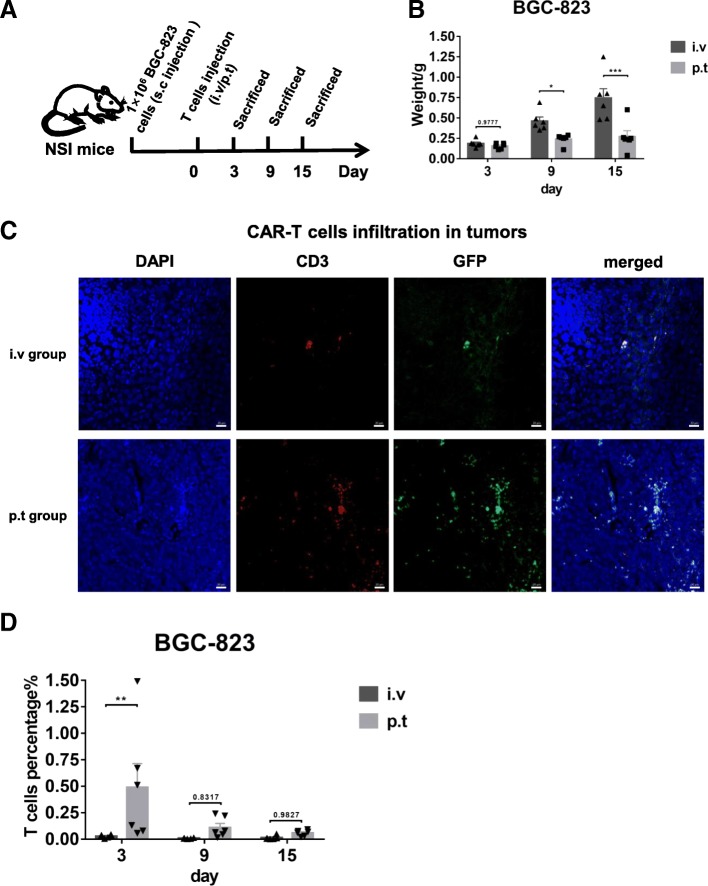
Infiltration of M28z10 T cells into GC tissue is enhanced by peritumoral delivery. **a** Schematic representation of the experiments. **b** Tumor weight of BGC-823 subcutaneously injected mice with different T cell delivery methods when sacrificed at each different time point. Error bars denote the s.e.m., and the results were compared with two-way ANOVA test. *P < 0.05; **P < 0.01; ***P < 0.001. **c** Representative Immunofluorescence imaging of the tumor tissue section for the BGC-823 s.c. mice with different T cell delivery methods sacrificed in day29, scale bar = 20 μm. **d** Detection of the percentage of tumor infiltrated T cells by FACS. Error bars denote the s.e.m., and the results were compared with two-way ANOVA test. *P < 0.05; **P < 0.01; ***P < 0.001

The improved efficacy of peritumoral delivery of M28z10 T cells is probably a result of the improved CAR-T cell infiltration into solid tumors in the early stage; to verify this, we again treated NSI mice bearing BGC-823 cells with M28z10 T cells via i.v. delivery or p.t. delivery and sacrificed them at different time point to examine the status of CAR-T cells infiltration (Fig. 7a). We detected T cells and CAR-T cells in the tumor tissue of these mice at three different time point after T cells injection by immunofluorescence imaging and FACS respectively. The results showed that the better suppressive effect of tumor growth could be observed from day 9 in p.t. group compared with i.v. group (Fig. 7b). More importantly, infiltration of T cells in the p.t. delivery group is higher than i.v. group as detected by immunofluorescence and FACS (Fig. 7c, d). Collectively, these findings demonstrated that M28z10 T cells can suppress GC tumor progression in the s.c. transplanted GC xenograft mouse models and peritumoral delivery approach can significantly improve CAR-T cells infiltration into solid tumor tissue in the early stage compared with intravenous delivery.

## Discussion

Targeted therapies, including small molecules, monoclonal antibodies, and adoptive cell transfer, have opened a new era of cancer treatment and significantly improved the prognosis of patients [39]. However, for a majority of GC patients, there is a lack of available targeted approaches, and the 5-year survival rate is far from satisfactory. Therefore, the development of targeted strategies is urgently needed to improve the clinical outcome of advanced GC patients and prolong their survival.

The applications of CAR T cells have achieved remarkable success in acute lymphoblastic leukemia. Moreover, many preclinical and clinical studies have suggested the potential of CAR T cells to treat solid cancer [33, 40]. Previously, we have showed the improved anti-tumor activities of the third-generation mesothelin targeting CAR T cells M28z10 compared with the second-generation M28z CAR T cells. Nonetheless, there have been few studies of CAR T cells targeting GC, which has limited their clinical usage. In the present study, we characterized MSLN as a target of human GC in multiple primary GC samples and cell lines and then generated third-generation CAR (M28z10) T cells to target MSLN+ GC cells. Our in vitro functional assays demonstrated that M28z10 T cells have significant antitumor activity against multiple MSLN+ GC cell lines and suppress GC progression in vivo, as evaluated in GC xenograft mouse models. Other groups have reported MSLN as a target of CAR T cells against multiple types of solid cancers. Zhao Y et al. reported that multiple injections of electroporated autologous mesothelin-targeted T cells can mediate regression of human ovarian cancer in NSG mice [41]. Beatty GL et al. reported the result of a phase 1 trial of mRNA electroporated mesothelin-specific chimeric antigen receptor T Cells to treat patients with pancreatic carcinoma metastases. This strategy was proved to be safe, and was shown to elicit anti-tumor responses [32]. However, mRNA electroporated CAR was only transiently expressed in T cells, so that the anti-tumor responses may not be sufficiently durable to elicit a remission. Interestingly, antigen spreading was observed in the case report published by the same group, suggesting the potential of CAR-mesothelin T cells to trigger the host intrinsic immune responses [42]. This may prompt us to combine CAR-T therapy with other immune stimulating adjuvants to promote tumor antigen presentation and recognition by host immune cells, thereby helping to overcome tumor antigen heterogeneity which is an obstacle for the current single target CAR-T immune therapy.

Although many studies have reported the efficacy of second- or third-generation CAR T cells against multiple types of solid cancer based on mouse models, clinical reports of complete remission in patients are very limited. This may be due to the T cell suppressive microenvironment in solid cancers and the large tumor burden in advanced patients, which limit the efficacy of current CAR T cell therapy [43, 44]. In our study, systemic i.v. administration of M28z10 T cells induced the regression of i.p.- and i.v.-transplanted diffuse GC tumors, but for the established BGC-823 s.c. tumors, the efficacy of i.v. M28z10 T cell treatment was modest at the same dosage, and peritumoral delivery strategy can result in improved CAR-T cells infiltration into tumor tissue in the early stage and cause tumor regression. Regional delivery of CAR-T cells can not only enhance the anti-tumor efficacy for injected tumors but also is believed to avoid the adverse effect caused by systemic delivery method [45–50]. Therefore, regional delivery is suitable to be used to inject anti-MSLN CAR T cells a clinical trial to minimize the risk of adverse effects in the future. Moreover, these data suggest that the antitumor activity of CAR T cells should be further enhanced by incorporating other functional elements into the CAR vectors to promote CAR T cell infiltration, to sustain T cell effector activities and to enhance cooperation with bystander T cells or innate immune cells [51, 52]. Current methods for implementing this strategy include the coexpression of immune-promoting cytokines [53–55], the secretion of scFv that blocks the PD-1 receptor [56, 57], and the introduction of dominant negative forms of inhibitory receptors with the CAR [52]. These modifications to our CAR T cells will be tested in future works to further improve the efficacy against large established gastric tumors.

Overall, we have demonstrated the feasibility and efficacy of M28z10 T cells against GC in vitro and in vivo in multiple xenograft mouse models. Our results suggest that mesothelin may be a potential target of CAR T cells for treating gastric cancer.

## Conclusions

In summary, we characterized mesothelin as an antigen of chimeric antigen receptor T cells in human gastric cancer, and utilized third-generation anti-Mesothelin CAR-T cell M28z10 to target human gastric cancer. Our data demonstrated that M28z10 T cells can exert potent anti-tumor activities in vitro and in vivo against GC as evaluated by several xenograft GC mouse models. These data suggest the potential value of employing M28z10 T cells to treat GC patients in the clinic.

## Abbreviations

BLI: Bioluminescence imaging
CAR: Chimeric antigen receptor
E:T: Effector to target
ELISA: Enzyme-linked immune absorbance assay
GC: Gastric cancer
GM-CSF: Granulocyte-macrophage colony-stimulating factor
GVHD: Graft versus host disease
i.p.: Intraperitoneally
i.v.: Intravenous
IFN-γ: Interferon-γ
IL-2: Interleukin-2
M28z10: a CAR containing the MSLN scFv, CD3ζ, CD28, and DAP10 intracellular signaling domain
MSLN: Mesothelin
NSI: NOD-SCID IL2Rγ^−/−^
p.t.: Peritumoral
PBMCs: Peripheral blood mononuclear cells
s.c.: Subcutaneous
scFv: Single-chain variable fragment
TAAs: Tumor-associated antigens
